# A Recognition Method for Road Hypnosis Based on Physiological Characteristics

**DOI:** 10.3390/s23073404

**Published:** 2023-03-23

**Authors:** Bin Wang, Huili Shi, Longfei Chen, Xiaoyuan Wang, Gang Wang, Fusheng Zhong

**Affiliations:** 1 College of Electromechanical Engineering, Qingdao University of Science & Technology, Qingdao 266000, China; 2 Collaborative Innovation Center for Intelligent Green Manufacturing Technology and Equipment of Shandong, Qingdao 266000, China

**Keywords:** advanced driver assistant system, physiological characteristics, road hypnosis, driver behavior

## Abstract

Road hypnosis is a state which is easy to appear frequently in monotonous scenes and has a great influence on traffic safety. The effective detection for road hypnosis can improve the intelligent vehicle. In this paper, the simulated experiment and vehicle experiment are designed and carried out to obtain the physiological characteristics data of road hypnosis. A road hypnosis recognition model based on physiological characteristics is proposed. Higher-order spectra are used to preprocess the electrocardiogram (ECG) and electromyography (EMG) data, which can be further fused by principal component analysis (PCA). The Linear Discriminant Analysis (LDA), Quadratic Discriminant Analysis (QDA), and K-Nearest Neighbor (KNN) models are constructed to identify road hypnosis. The proposed model has good identification performance on road hypnosis. It provides more alternative methods and technical support for real-time and accurate identification of road hypnosis. It is of great significance to improve the intelligence and active safety of intelligent vehicles.

## 1. Introduction

With the rapid development of the Chinese economy, Chinese car ownership continues to grow, especially that of private cars. The conflicts among people, vehicles, and the environment in the road traffic system are prominent. According to the research [1], the driver’s human factor is the main cause of road traffic accidents all over the world. The driver factor accounts for more than 70%. The physiological and psychological characteristics of drivers are the important factors that can affect driving behavior. These factors include gender, age, driving experience, personality, and driving propensity [2,3]. The driver’s dangerous driving behavior, such as distracted driving [4,5], driving fatigue [6,7], emotion [8,9], and road hypnosis, is closely related to these factors. Driver fatigue and distraction have been the main topics on which many scholars have conducted a lot of research. However, as a state of unconscious driving, road hypnosis is not the same as driver fatigue and distraction. It is usually accompanied by external features such as distracted attention, glazed eyes, and temporary memory loss. This phenomenon is more common in monotonous environments such as highways or tunnels. For example, in daily life, an experienced driver is easy to fall into a state of unconsciousness driving when they are driving on the highway for an hour or more. This is because the scene on both sides of the road is monotonous and repetitive, and straight driving is also a monotonous long-time task. Not only in highway scenarios, but also in highly predictable environments that are very familiar to drivers, road hypnosis is likely to occur. For instance, people who drive to work every day and commute for an hour are prone to road hypnosis when they drive on the same road day after day, year after year. When the driver falls into road hypnosis, although they can continue to drive normally, they do not have the alertness and reaction ability under normal conditions. Due to the high-speed driving of the car on the highway, it will be more harmful when an accident occurs. Therefore, it is meaningful to study road hypnosis in depth.

It has been shown in previous research that experienced drivers are more likely to fall into road hypnosis than novice drivers. A monotonous or familiar driving environment is also an important factor in inducing road hypnosis. Driver characteristics and road environment are two important factors affecting road hypnosis. Griffith et al. [10] made a clear distinction between hypnosis and sleep, and they first defined road hypnosis as a state of amnesia and trance. They believed that drivers can still drive normally in road hypnosis, but their reaction times were significantly slower. In subsequent studies [11], they further defined road hypnosis as a drowsy state with distorted thinking and cognition. The experiment was designed in the monotonic highway scene, and road hypnosis was induced by looking at two points. Shor et al. [12] believe that road hypnosis is a state of mental fatigue in which the driver is in a condition of fantasy and distorted thinking. The existence of road hypnosis was confirmed by the different reaction times of drivers to the ten inducement factors identified through the reaction time device. Wertheim et al. [13] offer a different view. They believe that the high predictability of the driving scene is the cause of road hypnosis. Cerezuela et al. [14] further corroborated this claim. They believe that the driver is in an unconscious driving mode under road hypnosis. A highly predictable environment was selected for the experiment. The driver was allowed to drive continuously for a long time to induce road hypnosis. Unlike previous researchers, they not only confirmed the existence of road hypnosis, but also attempted to identify it with relative parameters in statistics.

Compared with driving fatigue and driving distraction, the internal mechanisms and external manifestations of road hypnosis are different. Unlike driver fatigue, road hypnosis tends to occur intermittently and to a less degree during driving. Drivers experiencing road hypnosis are unaware that they experience it. They are unconscious for the duration of the driving. However, at the moment when a driver disengages from road hypnosis, it is usually accompanied by an alert state. Driver fatigue is a condition that lasts until the driver takes measures to overcome it. In contrast to driving distraction, road hypnosis and cognitive distraction are both states in which the driver’s attention diverts from driving. However, they are different. The cognitive resource attention of drivers in cognitive distraction is occupied by the secondary task, while that of drivers in road hypnosis are not. Road hypnosis is more likely to be affected by monotonous, repetitive, or familiar environments. It will appear and disappear repeatedly during driving. In essence, however, road hypnosis, driving fatigue, and driving distraction are all dangerous driving behaviors. Although the expressions and degrees are different, the physiological information of the driver in an abnormal state must be different from that in the normal state. At present, in the research of road hypnosis, physiological information has not been used to establish an effective identification model.

As a very important and effective impact in the study of driving behavior, driver physiological indicators have excellent performance in driver behavior recognition, especially in the study of driving fatigue and driving distraction. The contributions and research gaps of some related work are listed in Table 1.

Physiological characteristic indicators are selected to study road hypnosis of drivers in this paper. The identification model for road hypnosis is established with physiological data. The characteristic data of the driver in road hypnosis are collected by experiments, including the vehicle driving experiment and the simulated driving experiment. The identification model of road hypnosis is established by LDA [24], QDA [25], and KNN [26].

In our previous work [27], drivers’ eye movement characteristic parameter has been used to train the identification model for road hypnosis. Although road hypnosis can be accurately identified with this model, the interference caused by cognitive distraction cannot be excluded. For example, the same state of alertness occurs when normal drivers are stimulated by some kind of external disturbance. The driver may not have a relatively obvious external performance because the degree of stimulation is low or the driver has a strong psychological quality. This state can easily be mistaken for road hypnosis. Therefore, the experimental procedure is improved in this study. The parameter of eye movement is no longer used as the characteristic parameter but is used to judge whether road hypnosis occurs in the experiment to obtain the physiological characteristic parameter in road hypnosis. Compared with the experimental method in previous work, the characteristics parameters can be collected more accurately, and the accuracy of the model can be further improved with this method. The existence of road hypnosis is further demonstrated in this study. A method for identifying road hypnosis with different characteristic parameters is proposed. It provides more alternative methods and technical support for real-time and accurate identification of road hypnosis. It is of great significance to improve the intelligence and active safety of intelligent vehicles.

The contributions of this paper can be defined in the following three points:

(1) Design and organize the simulated driving experiment and the vehicle driving experiment, and collect physiological characteristic data of drivers under road hypnosis.
(2) Use KNN, LDA, and QDA algorithms to establish road hypnosis identification models. The identification model trained with KNN has the best performance in both the simulated driving experiment and the vehicle driving experiment.
(3) The existence of road hypnosis can be further confirmed in this study. It is proved that road hypnosis can be detected through a variety of different parameters.

## 2. Materials and Methods

### 2.1. Participants

**Figure 1:**
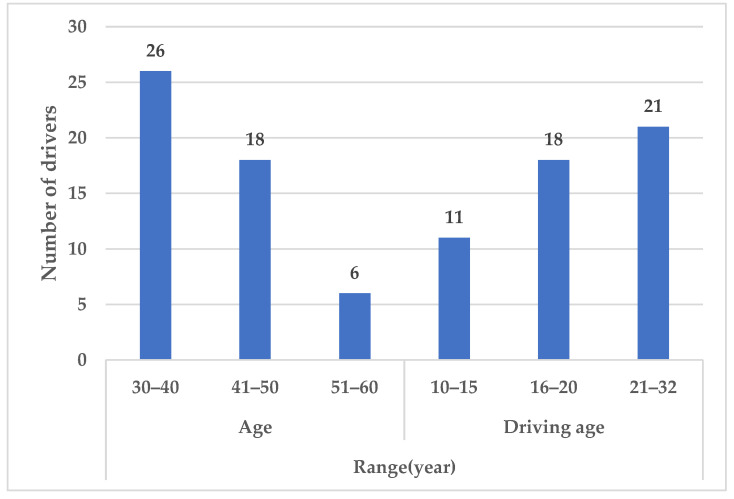
Basic information about drivers.

There are 50 participants, with a male-to-female ratio of 8:2. The basic information is shown in Figure 1. Participants are required to have no more than 600 degrees of myopia.

### 2.2. Equipment

The eye movement data is obtained using the eye tracker. Physiological information acquisition equipment is used to obtain the physiological characteristics of drivers. The drivers are paired with two laptops. The whole driving process is recorded on video.

**Figure 2:**
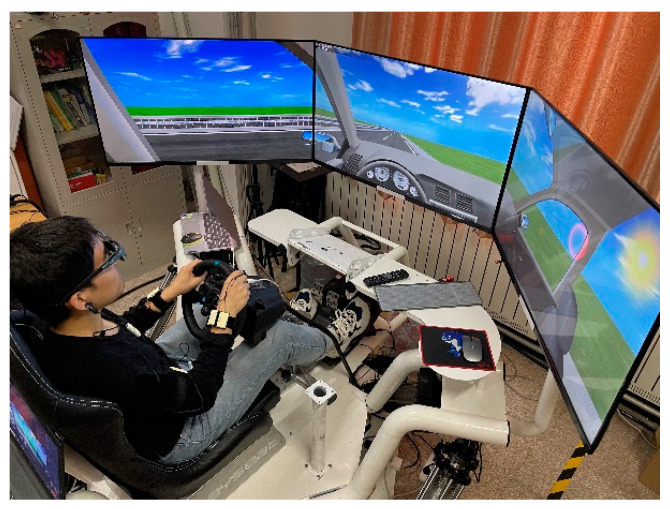
Simulated driving experiment environment.

A driving simulation system consisting of a Logitech G29 force feedback steering wheel, a pedal kit, a driving shift lever, a car seat, a six-degree-of-freedom platform, UC-win/Road software, and three 55-inch triage screens is also used in the simulation experiment. The simulated driving environment can be seen in Figure 2.

**Figure 3:**
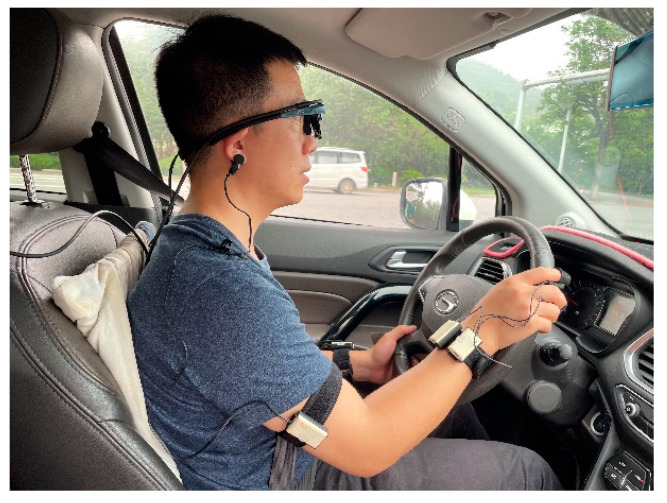
Vehicle driving experiment environment.

In the vehicle driving experiment, an SUV is used as the experimental vehicle, which is equipped with a power inverter to supply power to the equipment. The driving environment can be seen in Figure 3.

### 2.3. Procedure

#### 2.3.1. Simulated Driving Experiment

A monotonous highway scene is selected in the simulated driving experiment. A bi-directional four-lane highway, 40 km in length and 15 m in width, is generated in UC-win/Road software. There is no added curve throughout the road, all sections are straight. The participants drive back and forth on the scene until the end of the experiment. Participants get enough sleep to eliminate the disturbance caused by fatigue. The experiment starts at 9:00 a.m. and ends at 11:00 a.m. on weekdays. In addition to the participants, there are three experimental assistants involved. Before the start of the experiment, one assistant should debug the equipment, and two assistants should help the participant wear the eye tracker and physiological information acquisition equipment. In the experiment, the participant is required to drive at a speed of 120 km/h. is they are not allowed to change lanes during the course. A single driving process lasts 20 min. During the experiment, an assistant videotapes the entire driving process. At the same time, it is necessary to observe the driver’s eye movement with the eye tracker software in real time. The periods when the driver’s fixation point is directly ahead and barely pulsating are recorded. When the participant is likely to be alert during this period, an assistant immediately asks the participant if they have just experienced road hypnosis and then records it. At the end of a single driving process, the participant takes off the equipment and rests for ten minutes. An assistant checks the equipment. Another assistant asks the participant whether they experience any abnormal effects such as fatigue and distraction in the experiment and records it. The participant and assistants should watch the video playback of the experiment and the corresponding hotspot map. An assistant asks the participant again whether there is road hypnosis and records it. After the rest, the experiment is restarted. The procedure remains the same, but the experiment duration is changed to 40 min. When the driving process is complete, the assistants sort out the equipment and the experiment is ended.

#### 2.3.2. Vehicle Driving Experiment

As the driving speed on the highway scene is too fast to guarantee safety, the vehicle driving experiment is carried out in the tunnel. Qingdao Binhai Road is selected for the vehicle driving experiment. The start point is the Laoshan campus of Qingdao University of Science and Technology, and the end point is the Qingdao campus of Shandong University. The Laoshan campus of Ocean University of China is chosen as the intermediate point. The total length is 36 km. The speed limit is 80 km per hour. Both sides of the road have a more monotonous environment from the intermediate point to the end point. The participants pass through the Yangkou Tunnel with a length of 7.76 km. It consists of two left and right tunnels with six lanes. The length of the left tunnel is 3.875 km and that of the right tunnel is 3.888 km.

Due to traffic jams and other problems in the morning rush hour, the vehicle driving experiment starts at 10:00 a.m. and ends at 12:00 a.m. on weekdays. In addition to the participants, there are three assistants. The environment of the section from the start point to the intermediate point is complicated. Therefore, the participant is familiar with the equipment and environment of the vehicle in this section. After arriving at the intermediate point, a short rest is taken by the participant before continuing the experiment. Safety is the top priority for drivers during the experiment. The participant keeps driving at a constant speed and attempts to avoid overtaking and lane changing. During the experiment, an assistant videotapes the entire driving process. At the same time, it is necessary to observe the driver’s eye movement with the eye tracker software in real time. The periods when the driver’s fixation point is directly ahead and barely pulsating are recorded. When the participant is likely to be alert during this period, an assistant immediately asks the participant if they have just experienced road hypnosis and then record it. After arriving at the end point, the participant can take off the equipment and take a 20 min rest. An assistant checks the equipment. Another assistant asks the participant whether they experience any abnormal effects such as fatigue or distraction in the experiment and records it. The participant and assistants watch the video playback of the experiment and the corresponding hotspot map. An assistant asks the participant again whether they experience road hypnosis and records it. After the rest, the experiment is restarted. The participant drives back from the end point to the start point. The procedure remains the same. Once at the start point, the assistants arrange the equipment and finish the experiment.

## 3. Results

The experiment obtains 50 groups of vehicle data and 50 groups of simulated data after sorting and classifying. The data with typical road hypnosis is manually screened by colleagues with relevant research experience in the laboratory, which is used to make the road hypnosis data set. A total of 10 min of data is screened from each group of road hypnosis data. Since road hypnosis is a state of recurring and disappearing several times in a certain period, the 10 min of data screened is not a total period of continuous driving. Similarly, 10 min of data can be screened from normal driving data.

The data screening process is as follows. Firstly, 10 min of video footage from each set of experimental data is screened. The 10 min clip is not continuous, it is a patchwork of several video clips featuring road hypnosis. Six groups of experiments could not screen out video clips meeting the requirement of the clips being longer than 10 min. In other words, the six groups of experiments were excluded because the duration of road hypnosis in these experiments is less than 10 min. Therefore, 44 groups remained. That is why we only obtain 10 min of data for a long-time experiment. Secondly, nine participants in the vehicle experiment are fatigued due to physical exhaustion, uncomfortable sitting posture, and other reasons, so the nine groups of data are eliminated. Finally, 35 groups of valid video data are obtained as the data set of the vehicle driving experiment, including 25 groups of normal driving and 10 groups of road hypnosis.

In the simulated driving experiment, three groups were eliminated, and finally, 43 groups of effective video data were obtained, including 27 groups of normal driving and 16 groups of road hypnosis. No matter the data from the vehicle experiment or simulated experiment, 70% of the data is selected for calibration and training, and 30% of the data is selected for verification.

### 3.1. Data Pre-Processing and Feature Extraction

Raw signals of ECG and EMG were preprocessed using a Butterworth fourth-order filter [28] at 45 Hz and a fourth-order Chebyshev band pass filter [29] at 20–100 Hz, respectively. The specific calculation process is as follows.

A moment is a quantitative measure of a set of points, and a cumulate is a nonlinear combination of moments.

The first moment (mean) can be calculated by Formula (1):

(1) $$m_{1}=E\left[X\right].$$

The second moment (autocorrelation function) is as follows:

(2) $$m_{2}\left(\tau_{1}\right)=E\left[X\left(K\right)\cdot X\left(K+\tau_{1}\right)\right]=c_{2}\left(\tau_{1}\right).$$

Third moment is

(3) $$m_{3}\left(\tau_{1},\tau_{2}\right)=E\left[X\left(K\right)\cdot X\left(K+\tau_{1}\right)\cdot X\left(K+\tau_{2}\right)\right]=c_{3}\left(\tau_{1},\tau_{2}\right).$$

When the order is less than 4, the moment is equal to its cumulative moment, but this is not the case in the fourth order, as shown in Formulas (4) and (5).

Fourth moment:

(4) $$m_{4}\left(\tau_{1},\tau_{2},\tau_{3}\right)=E\left[X\left(K\right)\cdot X\left(K+\tau_{1}\right)\cdot X\left(K+\tau_{2}\right)\cdot X\left(K+\tau_{3}\right)\right],$$

(5) $$m_{4}\left(\tau_{1},\tau_{2},\tau_{3}\right)=c_{4}\left(\tau_{1},\tau_{2},\tau_{3}\right)+c_{2}\left(\tau_{1}\right)\cdot c_{2}\left(\tau_{3}-\tau_{2}\right)+c_{2}\left(\tau_{2}\right)\cdot c_{2}\left(\tau_{3}-\tau_{1}\right)+c_{2}\left(\tau_{3}\right)\cdot c_{2}\left(\tau_{2}-\tau_{1}\right).$$

The energy spectrum can be calculated by a second-order Fourier transform:

(6) $$P\left(f\right)=\sum_{\tau_{1}=-\infty }^{\infty }c_{2}\left(\tau_{1}\right)e^{-j\omega \tau_{1}}.$$

The Fourier transform of the third moment is called a bispectrum $B\left(f_{1},f_{2}\right)$:

(7) $$B\left(f_{1},f_{2}\right)=\sum_{\tau_{1}=-\infty }^{\infty }\sum_{\tau_{2}=-\infty }^{\infty }c_{3}\left(\tau_{1},\tau_{2}\right)e^{-j\left(\omega \tau_{1}+\omega \tau_{2}\right)}.$$

It can also be expressed using the Fourier transform:

(8) $$B\left(f_{1},f_{2}\right)=E\left[X\left(f_{1}\right)X\left(f_{2}\right)X*\left(f_{1}+f_{2}\right)\right].$$

Among this, $X\left(f\right)$ is the Fourier transform of the signal. ∗ is the conjugate operator. $E\left[*\right]$ is the expected operation.

The frequency f can be normalized between 0 and 1. The bispectrum given by Formula 8 is a complex-valued function of two frequencies. The Fourier transform conjugate of a real signal is symmetric, so the bispectrum is also symmetric. That is,

(9) $$B\left(f_{1},f_{2}\right)=B\left(f_{2},f_{1}\right)=B^{*}\left(-f_{1},-f_{2}\right)=B\left(-f_{1}-f_{2},f_{2}\right)=B\left(f_{1},-f_{1}-f_{2}\right)=B\left(-f_{1}-f_{2},f_{1}\right)=B\left(f_{2},-f_{1}-f_{2}\right).$$

In this work, we extract features from the bispectrum of the signal, resulting in a non-linear road hypnosis signature in ECG and EMG signals. Its different characteristics are as follows:

The sum of the logarithmic amplitudes of the bispectrum $S_{1}$:

(10) $$S_{1}=\sum_{\Omega }\log\left(\left|B\left(f_{1},f_{2}\right)\right|\right).$$

The sum of logarithmic amplitudes of bispectrality diagonal elements $S_{2}$:

(11) $$S_{2}=\sum_{\Omega }\log\left(\left|B\left(f_{k},f_{k}\right)\right|\right).$$

The first-order spectral moments of the amplitudes of the bispectral diagonal elements $S_{3}$:

(12) $$S_{3}=\sum_{k=1}^{N}k\log\left(\left|B\left(f_{k},f_{k}\right)\right|\right).$$

### 3.2. Fusion of ECG and EMG Features

Both ECG and EMG signals are capable of conveying different information when the driver experiences road hypnosis. The features obtained from only one of these two signals often only focus on some specific aspects, which may lead to inaccurate results. The driver’s state of road hypnosis is difficult to be observed directly but needs to be inferred from a variety of available information. For example, the ECG signal provides the heart rate information in road hypnosis, and the EMG signal provides the muscle fatigue or muscle action information in the road hypnosis state (especially when the driver leaves the road hypnosis state). While both signals can provide physiological information about drivers in road hypnosis, they can represent different parts of the physiological system. The results obtained by only focusing on the latent state information of certain physiological systems are unreliable. Therefore, combining ECG and EMG signals obtained from physiological systems can improve the identification accuracy for road hypnosis. In this study, ECG and EMG signals are fused with PCA [30]. Principal components are computed by determining the eigenvectors and eigenvalues of the covariance matrix. The covariance matrix is used to measure the degree of variation between dimensions from the mean. The covariance of two random variables is the trend of their change after fusion, calculated as follows:

(13) $$\mathrm{cov}\left(X:Y\right)=E\left[E\left[X\right]-X\right]\cdot E\left[E\left[Y\right]-Y\right].$$

Here, $E\left[X\right]$ and $E\left[Y\right]$ represent the expected values of X and Y, respectively. For the data obtained by the experimental sampling, it can be transformed into

(14) $$\mathrm{cov}\left(X:Y\right)=\sum_{i=1}^{N}\frac{\left(x_{i}-\bar{x}\right)\left(y_{i}-\bar{y}\right)}{N}.$$

N is the dimension of the data. $\bar{x}$ and $\bar{y}$ are the means of x and y. There are two components taken in experiments in the paper.

**Figure 4:**
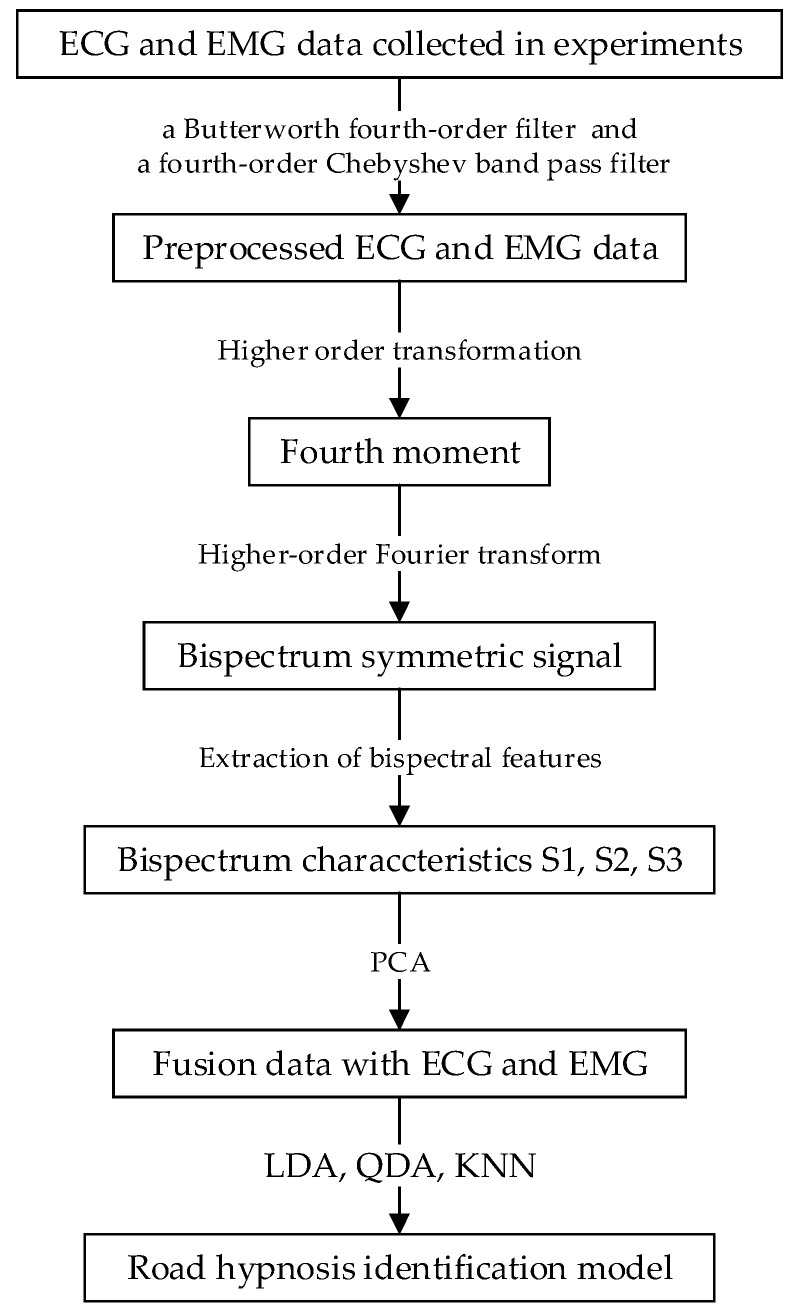
Process of establishing the road hypnosis identification model.

The process of establishing the road hypnosis identification model is shown in Figure 4.

### 3.3. Road Hypnosis Classification

We use the features extracted by LDA, QDA, and KNN for training and classification. Accuracy (AC), sensitivity (SE), and specificity (SP) are used to evaluate the models. In the training process, we tried to choose 5 to 10 for the value of K; the accuracy of K = 9 is the highest.

TP is the number of road hypnosis cases correctly identified in the data. TN is the number of normal driving cases. FP is the number of normal driving cases recognized as instances of road hypnosis. FN is the number of road hypnosis cases recognized as instances of normal driving.

Accuracy is the percentage of samples that are correctly identified, which can be calculated as follows:

(15) $$AC=\frac{TN+TP}{TN+TP+FN+FP}\times 100\%.$$

Sensitivity is the percentage of the number of road hypnosis samples correctly identified to the total number of road hypnosis samples. The higher the sensitivity, the lower the false positive rate. It can be calculated as follows:

(16) $$SE=\frac{TP}{FN+TP}.$$

Specificity is the percentage of the number of correctly identified normal driving samples to the total number of normal driving samples. If the specificity is higher, the misjudgment can be lower. It can be calculated as follows:

(17) $$SP=\frac{TN}{TN+FP}.$$

**Figure 5:**
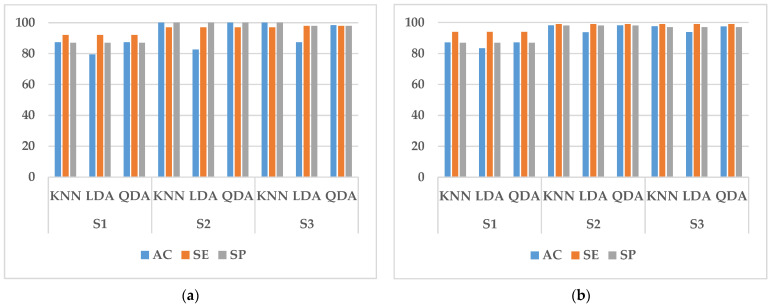
Random cross-validation of ECG data. (**a**) Vehicle driving experiment; (**b**) Simulated driving experiment.

**Figure 6:**
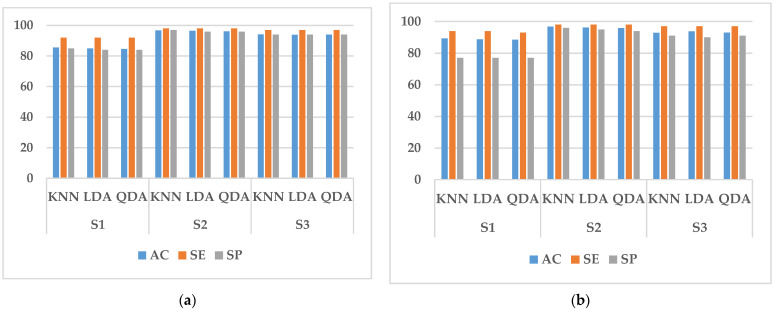
K-fold cross-validation of ECG data. (**a**) Vehicle driving experiment; (**b**) Simulated driving experiment.

**Figure 7:**
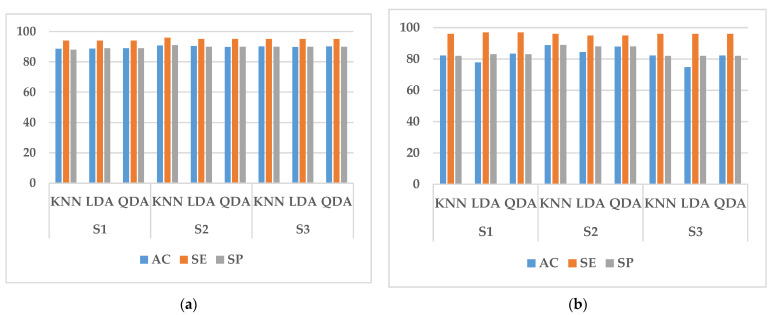
Random cross-validation of EMG data. (**a**) Vehicle driving experiment; (**b**) Simulated driving experiment.

**Figure 8:**
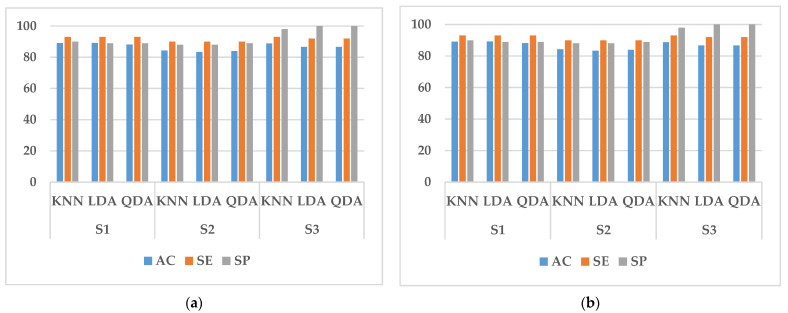
K-fold cross-validation of EMG data. (**a**) Vehicle driving experiment; (**b**) Simulated driving experiment.

**Figure 9:**
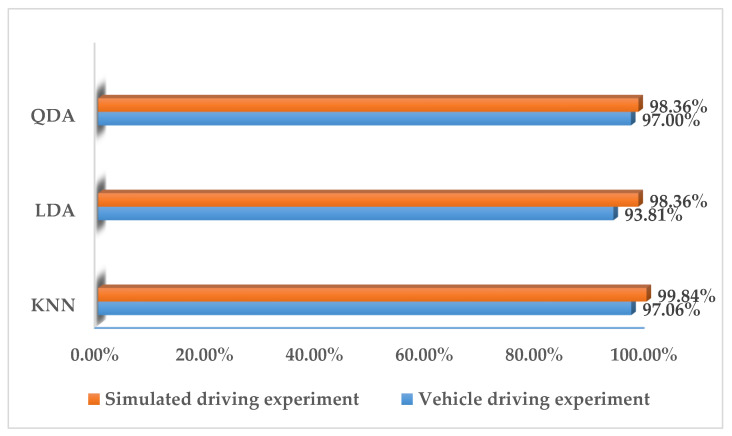
Accuracy of fusion with ECG and EMG signals.

In this study, we performed two types of validation, random cross-validation and k-fold cross-validation. In both cases, 70% of the data in the training set is used for training and 30% for testing. In randomized cross-validation, features extracted from all experimental data are shuffled, and training and testing datasets are randomly selected by the system. Although the performance is good, the main limitation is that it cannot be generalized. This is because the randomly assigned training and test datasets may belong to the same subject. When training and testing on data from the same subject, a common result is a large increase in the accuracy, sensitivity, and specificity of the classifier. To overcome it, k-fold cross-validation is conducted in this paper to estimate the generalization error. It can also be used for model selection, choosing one of several models with the smallest estimated generalization error. The advantage of this approach over random cross-validation is that all results are used for both training and testing, and each result is used only once in training and testing. The experimental results obtained are shown in Figure 5, Figure 6, Figure 7, Figure 8 and Figure 9.

## 4. Discussion

It can be seen from Figure 5, Figure 6, Figure 7 and Figure 8 that the experimental results of k-fold cross-validation are more reliable, and the values of the three parameters of accuracy, sensitivity, and specificity are more accurate. The accuracy of the three types of models established with the fused signals of ECG and EMG are shown in Figure 9. The identification model trained with KNN has the best performance in both the simulated driving experiment and the vehicle driving experiment. Compared with the vehicle driving experiment, the model of the simulated driving experiment has better performance. The reason is that the conditions of the simulated driving experiment are more controllable and there is less irrelevant interference, such as the sudden appearance of pedestrians and vehicles during driving. Therefore, the excitation effect of road hypnosis is better and has more typical characteristics. However, it is also shown in the results of the vehicle driving experiment that road hypnosis exists in the daily driving environment, especially in monotonous environments, and it can be identified with physiological characteristics.

Cognitive distraction is easy to interfere with the results due to similar external characteristics. However, cognitive distraction is not the same as road hypnosis, either the definition or the intrinsic mechanism. Cognitive distraction occurs when the cognitive resources of the driver are not focused on the main driving task. Although the cognitive resources of drivers in road hypnosis are not focused on the main driving task, they are also not occupied with other tasks. Therefore, no matter in the simulated driving experiment or the vehicle driving experiment, the method of inducing road hypnosis through secondary tasks is not adopted Because it is hard to completely separate road hypnosis from cognitive distraction with this method. We conducted naturalistic driving experiments without human intervention. The idea of our experiment is to make the participants drive the vehicle naturally in an environment where road hypnosis is easy to occur. By observing the changes in the driver’s eye movements during driving, the occurrence of road hypnosis can be determined through active inquiry. Therefore, the presence of cognitive dispersion is experimentally eliminated as much as possible. Similarly, participants are required to have a good rest before the experiment. After each experiment, the participant is immediately asked whether they have experienced fatigue or distraction. In addition, part of the data disturbed by fatigue or distraction is also eliminated during data screening. Therefore, no matter the part of the experimental method or data processing, we try our best to eliminate the interference caused by driving fatigue or distraction.

In our published work [27], eye-movement information is used to identify road hypnosis. In the previous study, the characteristic parameters of road hypnosis are determined by observing the ECG signals of drivers. The eye-movement information is collected for the identification of road hypnosis. However, it is susceptible to cognitive distraction or other distractions. For example, when the driver is normally driven by external stimuli, they also appear in an alert state. Because the degree of stimulation is low or the driver has a strong psychological quality, the driver may not have a relatively obvious external performance. This state can easily be mistaken for the alert state of road hypnosis. Therefore, in this study, we change the experimental procedure. The parameter of eye movement is no longer used to identify road hypnosis, but to observe and capture road hypnosis in the experiment to obtain the corresponding physiological characteristic parameters. Compared with the previous methods, it can more accurately collect the characteristic parameters when the driver experiences road hypnosis and further improve the accuracy.

Road hypnosis is a state in which the driver is unconscious. The driver is usually not subjectively aware of anything. However, at the moment of exiting this state, the driver is often in a distinct awakened state. That is the moment we ask the driver to determine whether road hypnosis occurs. This method can ensure an accurate record of road hypnosis. It can also make the driver avoid the interference brought by excessive inquiry. We do not choose to question the driver by watching the video replay after the experiment, even though it would have been better for the driver to keep driving naturally. The reason is that hypnosis is an unconscious driving state, and the accuracy of questioning afterward is low. The procedure in our experiment proved to be effective and reasonable.

## 5. Conclusions

In this paper, the physiological information of the driver in the natural driving process is collected by designing and carrying out a vehicle driving experiment and a simulated driving experiment for the driver in two typical monotonous scenarios of tunnel and expressway. The state of normal driving and road hypnosis can be identified. An identification model for road hypnosis based on physiological signals is presented. This paper mainly includes the following work:

(1) The experiments, including the simulated experiment and the vehicle experiment, are designed and organized. Fifty participants take part in the experiments. Experimental data including road hypnosis characteristic parameters are collected and screened. The physiological information database including simulated driving experiment data and vehicle driving experiment data is established for training, testing, and validation of the identification models.
(2) The data are preprocessed using a 45 Hz Butterworth fourth-order filter and a 20–100 Hz fourth-order Chebyshev band pass filter. Characteristic parameters are extracted from processed data by the high-order spectral feature method. The characteristic parameters of two different signals, ECG and EMG, are fused by principal component analysis and used as input parameters for the subsequently established road hypnosis identification model. Using the fusion feature parameters of ECG and EMG signals helps to make up for the lack of stability and poor robustness caused by a single physiological feature parameter when establishing a model.
(3) KNN, LDA, and QDA algorithms are used to establish road hypnosis identification models. Accuracy, sensitivity, and specificity are used to evaluate the models. It is shown through the experimental results that the models are effective. The existence of road hypnosis can be further confirmed in this study. It is proved that road hypnosis can be detected through a variety of different parameters. It plays an important role in improving the intelligence and safety of vehicles.

A few points in this research can be further studied and improved.

(1) The scientific experimental method and reasonable experimental procedure make the results almost free from the influence of cognitive distraction. However, the parameters in our experiment cannot directly distinguish road hypnosis and cognitive distraction. The interference with cognitive distraction has not been completely eliminated. It can be further attempted to select other physiological features such as EEG, which can be used to study the different characteristics of drivers between road hypnosis and cognitive distraction.
(2) To be in a natural driving state, the participant does not need to complete a specific task during the experiment. However, active questioning to determine the occurrence of road hypnosis inevitably produces some interference. In further research, external characteristics can be explored to determine the emergence of road hypnosis.
(3) There are only monotonous scenes including tunnels and highways in our experiment. Road hypnosis also tends to occur in highly predictable environments, such as the familiar road environment of a particular driver’s daily commute.
(4) Due to the lack of closed highway test sections, no vehicle driving verification on a highway is conducted. In the follow-up study, we will apply for the use of closed highway sections for vehicle driving experiments.
(5) Many sensors used in the research have not been used in a mass-produced car. Not only is the device inconvenient to wear and interferes with the normal driving process, but it is also expensive and difficult to achieve mass production. This leads to a reduction in effectiveness of the proposed methods. However, it is often an essential part of research. This experimental equipment, which cannot be used in mass-produced cars, can collect more comprehensive and accurate parameters to reveal the mechanism of driving behavior. Therefore, it is demonstrated that it is feasible to use these parameters to identify these driving behaviors, and relevant methods are provided as technical support. For example, we have previously tried using cameras to identify driver fatigue [31] and distraction [32], which is the work conducted on the basis of our research and understanding of driving fatigue and driving distraction. This study is an exploratory study. The purpose is to explore the feasibility of using physiological parameters to identify road hypnosis. In future research, other sensors should be considered to explore a more effective method to identify road hypnosis in mass-produced cars.

## Figures and Tables

**Table 1:** Some researches on physiological signal identification of dangerous driving behavior.

Author	Contributions	Gaps
Pinto et al. [15]	Once the driver feels fatigued, their heart rate variability (HRV) value changes significantly.	It is expected that with the integration of robust template or model update techniques, the results would experience further improvements.
Habibifa et al. [16]	Four physiological signals are used to detect negative driver emotions, including ECG, EMG, electrodermal (EDA), and electroencephalogram (EEG). The results show that using ECG and EDA to detect negative emotions obtains more accurate results.	A small number of features for each biological signal is used in this research. Further studies may consider the four biological signals with other information such as environmental conditions, traffic, time, and so forth.
Hu [17]	The fuzzy entropy, sample entropy, and spectral entropy of the driver’s EEG signal are extracted through a driving simulator. These three EEG feature vectors are input into the Ada-boost classifier, and the results showed that the contribution rate of fuzzy entropy to the fatigue identification model is maximum.	There are similarities between the feature parameters used, and the performance of the model has not been verified in the vehicle driving environment.
Du et al. [18]	The local feedback fuzzy neural network (FNN) is used to process the EEG signal. The processed parameters are input into the TSK convolutional recursive fuzzy network (TCRFN) to identify the driver’s fatigue state. Experimental results show that the accuracy of the model in detecting fatigue driving of 19 test subjects is 88%.	The impact of EEG signals of different subjects on the proposed method needs to be further addressed in the future. A reasonable compromise between denoising operations and the prediction accuracy of the proposed model needs to be considered in the future.
Zhang et al. [19]	A clustering algorithm is used to extract the ECG space nodes of different drivers’ EEG signals. A pulse-coupled neural network is used to identify different degrees of fatigue.	The model has not been validated and evaluated on the test sample.
Murugan et al. [20]	Fatigue states are divided into four categories, which are sleepiness, inattention, fatigue, and cognitive inattention. The characteristic parameters such as the driver’s heart rate and HRV are extracted through ECG sensors. The selected features are trained using support vector machine (SVM), K-nearest neighbors (KNN), and integrated algorithms. The model performs well in the classification of single fatigue.	The identification accuracy of the four-category fatigue state detection integrated algorithm is only 58.3%.
Ramos et al. [21]	Nine main features are extracted from EMG and HRV to build an SVM classifier to identify the driver’s fatigue.	A future implementation could benefit from the real-time algorithm.
Barua et al. [22]	The effect of adding contextual information to the physiological features improved the classification accuracy by 4% in multiclass classification and by 5% in binary classification.	More contextual information can be included in the system. Classifiers can be adapted to the current driver as new unlabeled data.
Martensson et al. [23]	A portable digital recording system is used to extract the driver’s EEG, ECG, and electrooculogram (EOG) signals. The dimensionality reduction of feature parameters is carried out with the sequential floating forward selection (SFFS) algorithm. The selected parameters are trained by Random Forest (RF) model. The verification results show that the accuracy can reach 94.1%.	The model cannot perform well on the vehicle driving dataset.

## Data Availability

The data presented in this study are available on request from the corresponding author. The data are not publicly available due to privacy.

## References

[1] Adanu E.K., Smith R., Powell L., Jones S. (2017). Multilevel analysis of the role of human factors in regional disparities in crash outcomes. Accid. Anal. Prev..

[2] Wang X.Y., Liu Y.Q., Guo Y.Q., Xia Y.Y., Wu C.Z. (2019). Transformation mechanism of vehicle cluster situations under dynamic evolution of driver’s propensity. Transp. Res. F.

[3] Wang X.Y., Liu Y.Q., Wang J.Q., Zhang J.L. (2019). Study on influencing factors selection of driver’s propensity. Transp. Res. D.

[4] Kaber D., Jin S., Zahabi M., Pankok C. (2016). The effect of driver cognitive abilities and distractions on situation awareness and performance under hazard condition. Transp. Res. F.

[5] Alexey K., Roman S., Christian K., Alexander S. (2021). Driver distraction detection methods: A literature review and framework. IEEE Access.

[6] Yan Y., Yuan H.Z., Yang X.L., Liu G., Guo Z.Y., Wang L. (2021). A model of the relationship between monotonic road environment and driving fatigue based on multi-source data. China J. Highw. Transp..

[7] Salvati L., d’Amore M., Fiorentino A., Pellegrino A., Sena P., Villecco F. (2021). On-road detection of driver fatigue and drowsiness during medium-distance journeys. Entropy.

[8] Wang X.Y., Liu Y.Q., Wang F., Wang J., Liu L., Wang J. (2019). Feature extraction and dynamic identification of drivers’ emotions. Transp. Res. F.

[9] Liu Y.Q., Wang X.Y. (2023). The analysis of driver’s behavioral tendency under different emotional stated based on a Bayesian Network. IEEE Trans. Affect. Comput..

[10] Griffith W.W. (1963). Highway hypnosis: An hypothesis. Int. J. Clin. Exp. Hyp..

[11] Griffith W.W., Shor R.E. (1970). An historical note on highway hypnosis. Accid. Anal. Prev..

[12] Shor R.E., Thackray R.I. (1970). A program of research in “highway hypnosis”: A preliminary report. Accid. Anal. Prev..

[13] Wertheim A.H. (1978). Explaining highway hypnosis: Experimental evidence for the role of eye movements. Accid. Anal. Prev..

[14] Cerezuela G.P., Tejero P., Choliz M., Chisvert M., Monteagudo M.J. (2004). Wertheim’s hypothesis on ‘highway hypnosis’: Empirical evidence from a study on motorway and conventional road driving. Accid. Anal. Prev..

[15] Pinto J.R., Cardoso J.S., Lourenco A. (2017). Towards a continuous biometric system based on ECG signals acquired on the steering wheel. Sensors.

[16] Habibifa N., Salmanzadeh H. (2022). Improving driving safety by detecting negative emotions with biological signals: Which is the best?. Transp. Res. Rec..

[17] Hu J. (2017). Automated detection of driver fatigue based on AdaBoost classifier with EEG signals. Front. Comput. Neurosci..

[18] Du G., Wang Z., Li C., Liu P.X. (2021). A TSK-Type convolutional recurrent fuzzy network for predicting driving fatigue. IEEE Trans. Fuzzy Syst..

[19] Zhang C., Sun L., Cong F., Kujala T., Ristaniemi T., Parviainen T. (2020). Optimal imaging of multi-channel EEG features based on a novel clustering technique for driver fatigue detection. Biomed. Signal Process..

[20] Murugan S., Selvaraj J., Sahayadhas A. (2020). Detection and analysis: Driver state with electrocardiogram (ECG). Phys. Eng. Sci. Med..

[21] Ramos G., Vaz J.R., Mendonca G.V., Pezarat C.P., Rodrigues J., Alfaras M., Gamboa H. (2020). Fatigue evaluation through machine learning and a global fatigue descriptor. J. Healthc. Eng..

[22] Barua S., Ahmed M.U., Ahlstrom C., Begum S. (2019). Automatic driver sleepiness detection using EEG, EOG and contextual information. Expert Syst. Appl..

[23] Martensson H., Keelan O., Ahlstrom C. (2018). Driver sleepiness classification based on physiological data and driving performance from real road driving. IEEE Trans. Transp. Syst..

[24] Blei D.M., Ng A.Y., Jordan M.I. (2003). Latent dirichlet allocation. J. Mach. Learn. Res..

[25] Srivastava S., Gupta M.R., Frigyik B.A. (2007). Bayesian quadratic discriminant analysis. J. Mach. Learn. Res..

[26] Peterson L.E. (2009). K-nearest neighbor. Scholarpedia.

[27] Shi H.L., Chen L.F., Wang X.Y., Wang B., Wang G., Zhong F.S. (2023). Research on recognition of road hypnosis in the typical monotonous scene. Sensors.

[28] Zhang C.C., Shang L., Wang Y.K., Tang L. (2020). A CMOS programmable fourth-order butterworth active-RC low-pass filter. Electronics.

[29] Wang K., Wong S.W., Sun G.H., Chen Z.N., Zhu L., Chu Q.X. (2015). Synthesis method for substrate-integrated waveguide bandpass filter with even-order chebyshev response. IEEE Trans. Comp. Pack. Man..

[30] Abdi A., Willams L.J. (2010). Principal component analysis. Wires Comput. Stat..

[31] Wang X., Chen L., Zhang Y., Shi H., Wang G., Wang Q., Han J., Zhong F. (2022). A real-time driver fatigue identification method based on GA-GRNN. Front. Public Health.

[32] Shi H., Chen L., Wang X., Wang G., Wang Q. (2022). A nonintrusive and real-time classification method for driver’s gaze region using an RGB camera. Sustainability.

